# The Occurrence and Co-Occurrence of Regulated, Emerging, and Masked Mycotoxins in Rice Bran and Maize from Southeast Asia

**DOI:** 10.3390/toxins14080567

**Published:** 2022-08-19

**Authors:** Wipada Siri-anusornsak, Oluwatobi Kolawole, Warapa Mahakarnchanakul, Brett Greer, Awanwee Petchkongkaew, Julie Meneely, Christopher Elliott, Kanithaporn Vangnai

**Affiliations:** 1 Department of Food Science and Technology, Faculty of Agro-Industry, Kasetsart University, Bangkok 10900, Thailand; 2 Institute for Global Food Security, School of Biological Science, Queen’s University, Belfast BT9 5DL, UK; 3 The International Joint Research Center on Food Security, 113 Thailand Science Park, Phahonyothin Road, Pathum Thani 12120, Thailand; 4 School of Food Science and Technology, Faculty of Science and Technology, Thammasat University, Pathum Thani 12120, Thailand; 5 Center of Excellence in Food Science and Innovation, Thammasat University, Pathum Thani 12120, Thailand

**Keywords:** multi-mycotoxin, contamination, survey, feed, LC-MS/MS, Southeast Asia

## Abstract

Raw feed materials are often contaminated with mycotoxins, and co-occurrence of mycotoxins occurs frequently. A total of 250 samples i.e., rice bran and maize from Cambodia, Laos, Myanmar, and Thailand were analysed using state-of-the-art liquid chromatography-mass spectrometry (LC-MS/MS) for monitoring the occurrence of regulated, emerging, and masked mycotoxins. Seven regulated mycotoxins – aflatoxins, ochratoxin A, fumonisin B_1_, deoxynivalenol, zearalenone, HT-2, and T-2 toxin were detected as well as some emerging mycotoxins, such as beauvericin, enniatin type B, stachybotrylactam, sterigmatocystin, and masked mycotoxins, specifically zearalenone-14-glucoside, and zearalenone-16-glucoside. *Aspergillus* and *Fusarium* mycotoxins were the most prevalent compounds identified, especially aflatoxins and fumonisin B_1_ in 100% and 95% of samples, respectively. Of the emerging toxins, beauvericin and enniatin type B showed high occurrences, with more than 90% of rice bran and maize contaminated, whereas zearalenone-14-glucoside and zearalenone-16-glucoside were found in rice bran in the range of 56–60%. Regulated mycotoxins (DON and ZEN) were the most frequent mycotoxin combination with emerging mycotoxins (BEA and ENN type B) in rice bran and maize. This study indicates that mycotoxin occurrence and co-occurrence are common in raw feed materials, and it is critical to monitor mycotoxin levels in ASEAN’s feedstuffs so that mitigation strategies can be developed and implemented.

## 1. Introduction

Animal feed is an important part of the food chains and shows an enormous role in the safety and quality of animal by-products (meat products and dairy products) in the food supply chain [1]. However, natural contamination of feed raw materials with fungal pathogens, both before and after harvest, is a continuing and growing problem worldwide as many of these fungal species produce toxic metabolites (known as mycotoxins) that can profoundly affect animal health [2,3].

Rice bran and maize are common feed ingredients used by ASEAN’s feed industry, especially for poultry and swine. Proportions of 10–30% rice bran and 48–60% maize are recommended for animal feed to guarantee suitable nutrient contents. Rice bran is an abundant rice by-product of rice grain milling, especially during rice harvest seasons. ASEAN rice production in 2020 (the crop year 2019/2020) was 187.49 million tons, with rice by-products reaching 20.62 million tons annually [4]. However, rice bran is frequently contaminated with mycotoxins because of the proliferation of toxin-producing fungi at the aleurone layer [5]. In a previous survey of rice bran, detectable levels of mycotoxins, especially those produced by *Aspergillus* genera [6,7,8] were found, with aflatoxins (AFs) and ochratoxin A (OTA) found at concentrations of up to 28,000 µg·kg^−1^ and 12,000 µg·kg ^−1^, respectively [8].

In comparison, ASEAN maize production in 2020 (the crop year 2019/2020) was 44.94 million tons [4]. The ratio of maize output to domestic usage in ASEAN was expected to be 76.46%, with most maize production used for animal feed. Myanmar is the biggest maize exporter in ASEAN while Thailand is the major importing country of maize in the region from Myanmar, Cambodia, and Laos [4,9]. Maize is most commonly contaminated with mycotoxins during production and storage. Aflatoxins, fumonisins (FBs), deoxynivalenol (DON), and zearalenone (ZEN) are the most common mycotoxins found in maize worldwide and are often contaminated simultaneously with various other mycotoxins from *Fusarium* and *Aspergillus* species [2,6,10,11]. 

Mycotoxins in feed, particularly rice bran and maize, are a serious health concern in animals and humans that must be controlled and minimized. Dietary exposure of farm animals to mycotoxins has a detrimental impact on their health, performance, and productivity, with the severity and observed symptoms dependent on the type of mycotoxin present, concentration, and duration of exposure, gender, age, type of animal and exposure to other feed contaminants [3,12]. Additionally, chronic exposure of livestock animals to feed contaminated with mycotoxins can also cause the accumulation of mycotoxin residues in tissues and organs of animals in addition to carry-over of mycotoxins to animal-derived food products (meat, dairy, and eggs), leading to the indirect human intake of mycotoxins [13,14]. Mycotoxins are a frequent occurrence and their negative effects on human and animal health. Several countries have established mycotoxin regulations for food and feed. In the European Union (EU), animal feed products have been regulated for AFB_1_ by legislation through the European Commission Directive 2002/32/EC [15] and limits have been set for DON, OTA, ZEN, T-2 toxin (T-2), HT-2 toxin (HT-2), fumonisin B_1_ (FB_1_) and fumonisin B_2_ (FB_2_) [16] as shown in Table S1.

Of the hundreds of mycotoxins identified to date, regulated mycotoxins are considered the most frequently found feed contaminants that can potentially induce subacute and acute toxic effects in farmed animals and, therefore, have been regulated in many parts of the world [17,18]. Nevertheless, frequently other mycotoxins are found in feedstuffs; the so-called emerging mycotoxins, as well as plant-derived conjugates known as masked mycotoxins, are not regulated because of their low prevalence and toxicological facts [19].

The occurrence of new and rising combinations of mycotoxins in food and feed shows the capacity of fungi to adapt to changing environmental conditions [20]. Therefore, it is critical to understand the environmental factors and biodiversity of mycotoxigenic fungi that can produce mycotoxins and contaminate agricultural crops. Changes in climatic conditions, i.e., increased temperatures and altered rainfall patterns, have been identified as major drivers of fungal growth and contamination of agricultural crops relating to food/feed safety [21,22,23,24]. In the widely used Climate Risk Index by Germanwatch, Myanmar and Thailand were highlighted as being the countries most affected by extreme weather events between 2000–2019 followed by Cambodia and Laos [25]. Thus, there is a need for continuous surveillance in the future and constant review of mycotoxin risk management to ensure feeds are secure in the food chain and compliant with legislation and regulation requirements. A recent global feed survey shows that the combined feed output of the 10 ASEAN nations is projected to reach more than 110 million metric tons by 2025, making the ASEAN-10 one of the world’s largest producers of animal feed after the USA, China, and Russia [26]. However, due to poor agricultural practices, changing climatic conditions, and high dependence on imported cereals and other raw materials for feed production from the international market, there is a risk of a high prevalence of mycotoxins in feed and feed ingredients in this region [27]. 

Regulated mycotoxins are routinely analysed and monitored by the ASEAN national laboratories; however, there is a limited analytical testing capability and regulatory framework for the other important mycotoxins in feed raw materials [27]. Moreover, there is a lack of data available regarding multiple mycotoxins in feed ingredients. Therefore, monitoring is crucial to determine the exposure of livestock animals to multiple mycotoxins and evaluate the compliance of animal feed raw materials in this region to regulatory requirements. This study aimed to ascertain the prevalence of mycotoxins and their natural co-occurrence in raw feed materials collected across various local feed mills in four ASEAN countries- Cambodia, Laos, Myanmar, and Thailand- using state-of-the-art liquid chromatography-mass spectrometry (LC-MS/MS). Samples were analysed for the occurrence of the regulated mycotoxins (aflatoxins, deoxynivalenol, fumonisin B_1_ (FB_1_), HT-2, T-2 toxin, ochratoxin A, zearalenone), emerging mycotoxins (alternariol (AOH), beauvericin (BEA), enniatin type A (ENN A and A_1_), enniatin type B (ENN B and B_1_), ergocornine (Ergocor), ergocristine (Ergocris), stachybotrylactam (STLAC), sterigmatocystin (STC)), and masked mycotoxins (3-acetyl deoxynivalenol (3-Acetyl-DON), 15-acetyl deoxynivalenol (15-Acetyl-DON), deoxynivalenol-3-glucoside (DON-3-Glu), alpha-zearalenol (α-ZEN), beta-zearalenol (β-ZEN), zearalenone-14-glucoside (ZEN-14-G) and zearalenone-16-glucoside (ZEN-16-G)). These fungal metabolites are natural products produced by several *Aspergillus*, *Fusarium*, and *Penicillium* species. Emerging and masked mycotoxins are most commonly observed in food and feed [28,29,30], which may result from the lack of good agricultural practices (GAPs), good manufacturing practices (GMPs), hazard analysis, and critical control points (HACCP) [31]. However, the occurrence data of these mycotoxins are still limited. Thus, the data generated will be used to inform research efforts in ASEAN on the toxicological effects of fungal metabolites.

## 2. Results

### 2.1. Method Performance 

The multi-analyte method was validated based on the guidelines recommended by European Commission Regulation No. 2002/657/EC and SANTE/12089/2016. The coefficients of determination (R^2^) for the tested mycotoxins were >0.990, showing good linearity for the calibration curves. Limits of detection (LOD) and limits of quantification (LOQ) for the compounds analysed ranged from 0.05–25 µg·kg^−1^ and 0.5–50 µg·kg^−1^, respectively, for rice bran, while for maize LODs and LOQs ranged from 0.04–25 µg·kg^−1^ and 1–50 µg·kg^−1^, respectively. The trueness and the precision (RSD%) of the method were satisfactory for all the studied compounds. In rice bran, RDSs of ≤13% and ≤15% were observed for repeatability (RSDr) and reproducibility (RSDR), respectively, for the target mycotoxins. In maize, RSD values of ≤15% and ≤14% for repeatability and reproducibility, respectively, were found. The method performance is shown in Tables S2 and S3.

The performance characteristics of the multi-analyte method were within the acceptable range [15,16], indicating that the method could be applied successfully to detect and quantify low concentrations of mycotoxins in feed raw materials. The LODs and LOQs for target compounds in our method were much lower than the maximum acceptable levels of regulated mycotoxins in cereal and cereal products (rice bran) and maize [15,16].

### 2.2. Occurrence of Mycotoxins in Different Commodities

A total of 125 rice bran samples and 125 maize samples were analysed to determine contamination levels of mycotoxins. Regulated mycotoxins, including AFs, DON, FB_1_, HT-2, T-2, OTA, and ZEN, were observed as well as emerging mycotoxins and masked mycotoxins (Table S4). The emerging mycotoxins; AOH, BEA, ENN B, ENN B_1_, STLAC, STC, and masked mycotoxins; ZEN-14-G and ZEN-16-G were detected in rice bran and maize samples (Table S4). ENN A_1_, 15-Acetyl-DON, and DON-3-Glu were absent in rice bran and maize samples. On the other hand, ENN A, Ergocor, and Ergocris levels were below 2.5 µg·kg^−1^ (data not shown) in both rice bran and maize samples. The prevalence percentage of mycotoxin was calculated by the ratio of positive samples to total samples multiplied by 100. The prevalence of mycotoxins in rice bran was higher than in maize, with most samples indicating the presence of multiple mycotoxins, up to 7 mycotoxins, in rice bran samples.

#### 2.2.1. Rice Bran

**Figure 1:**
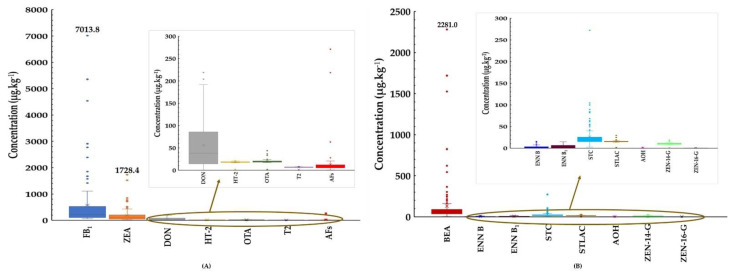
Boxplots showing mycotoxin concentrations in rice bran. (**A**) Contamination levels of regulated mycotoxins in rice bran. (**B**) Contamination levels of non-regulated mycotoxins in rice bran.

A total of 125 rice bran samples were analysed for the presence the regulated mycotoxins i.e., AFs (AFB_1_, AFB_2_, AFG_1_, AFG_2_), FB_1_, ZEN, OTA, DON, HT-2, and T-2; emerging mycotoxins including BEA, ENN B, ENN B_1_, STC, and STLAC and masked mycotoxins (ZEN-14-G and ZEN-16-G) as shown in Figure 1.

All rice bran samples were found to be contaminated with AFs, FB_1_, and ZEN, and the observed maximum concentrations were 271.1 µg·kg^−1^, 7013.8 µg·kg^−1^, and 1728.4 µg·kg^−1^, respectively. OTA and DON were prevalent in rice bran at 99% (124 of 125) and 94% (117 of 125), with maximum concentrations of 43.7 µg·kg^−1^ and 218.9 µg·kg^−1^, respectively (Figure 1 and Table S4). The most heavily contaminated samples by AFs, FB_1,_ and ZEN were those sourced in Myanmar. OTA and DON contamination were found in Laos and Thai samples, respectively.

Emerging mycotoxins -BEA, ENN B, and ENN B_1_ were observed across all samples (100%), with STC found in 98% (122 of 125) of rice bran samples. The maximum concentrations of BEA, ENN B, ENN B_1_, and STC were 2281.0 µg·kg^−1^, 15.4 µg·kg^−1^, 14.7 µg·kg^−1^, and 272.3 µg·kg^−1^, respectively (Figure 1 and Table S4). Beauvericin was prevalent in the Myanmar samples, while widespread contamination with ENN B and STC was observed in Thai samples. ENN B_1_ was the most frequently emerging mycotoxin found in Laos samples.

The masked mycotoxins contaminating rice bran were ZEN-14-G and ZEN-16-G and occurred in 60% (75 of 125) and 56% (70 of 125) of the samples at maximum concentrations of 20.7 µg·kg^−1^ and 39.5 µg·kg^−1^, respectively (Figure 1 and Table S4). Only one sample collected from Thailand contained 3-Acetyl-DON at 961.9 µg·kg^−1^ (data not shown). In this study, 113 samples (90.4%) were co-contaminated with major and emerging mycotoxins, with some samples indicating the presence of 4 to 7 mycotoxins.

#### 2.2.2. Maize

**Figure 2:**
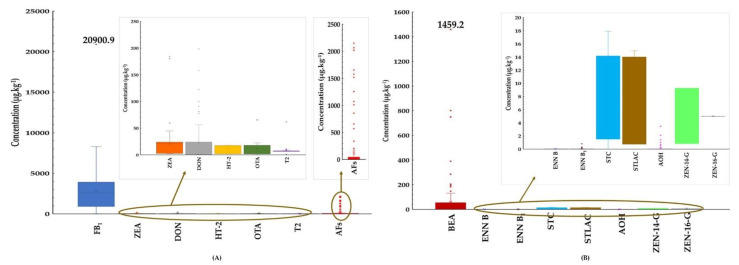
Boxplots showing mycotoxin concentrations in maize. (**A**) Contamination levels of regulated mycotoxins in maize. (**B**) Contamination levels of non-regulated mycotoxins in maize.

The regulated mycotoxins (AFs, FB_1_, ZEN, OTA, DON, HT-2, and T-2); emerging mycotoxins (BEA, ENN B, ENN B_1_, STC, and STLAC), and masked mycotoxins (ZEN-14-G and ZEN-16-G) were all detected in maize samples. The data were summarised and presented in boxplots (Figure 2).

The most frequently regulated mycotoxins were AFs, detected in 100% of the samples with a maximum concentration of 2149.7 µg·kg^−1^. FB_1_ and OTA contaminated 97% (121 of 125) and 90% (113 of 125) of the samples with maximum concentrations of 20,900.9 µg·kg^−1^ and 65.5 µg·kg^−1^, respectively (Figure 2 and Table S4). Widespread contamination by AFs, FB_1,_ and OTA was found in Laos, Myanmar, and Thai samples, respectively. Only one sample collected from Thailand contained HT-2 toxin at 9786 µg·kg^−1^.

The emerging mycotoxins BEA, ENN B, and ENN B_1_ were detected in 93–99% of the samples at maximum levels of 1459.2 µg·kg^−1^, 0.03 µg·kg^−1^, and 0.8 µg·kg^−1^, respectively (Figure 2 and Table S4). Moreover, BEA was reported in high concentrations in samples from Myanmar and Thailand, while ENN type B, detected in Thai, Myanmar, Cambodia, and Laos samples, all showed similar concentrations and frequency of contamination.

The prevalence of the masked mycotoxins, ZEN-14-G and ZEN-16-G, indicated that less than 3% of maize samples were contaminated with these. In this study, the 51 positive maize samples (40.8%) were co-contaminated with both regulated and emerging mycotoxins, with some samples indicating the co-occurrence of five mycotoxins.

### 2.3. Co-Occurrence of Mycotoxin Contamination

**Figure 3:**
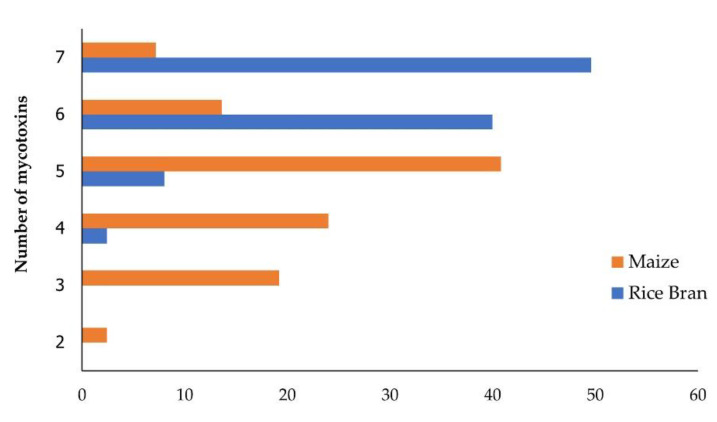
Number of regulated mycotoxins detected in rice bran and maize.

This study found the co-occurring regulated mycotoxins (AFs, DON, FB1, T-2, HT-2, OTA, and ZEN) combinations in rice bran and maize samples. The positive samples (100%) were simultaneously contaminated with at least two mycotoxins. Rice bran samples were contaminated with four, five, six, and seven mycotoxins at 2.4%, 8.0%, 40.0%, and 49.6%, respectively, and the co-occurrence of 7 mycotoxins was observed in all four countries. Multiple mycotoxins were detected in maize at 2.4%, 19.2%, 24.0%, 40.8%, 13.6%, and 7.2% for two, three, four, five, six, and seven mycotoxins, respectively (Figure 3).

The co-occurrence of regulated mycotoxins was found in rice bran and maize. The greatest co-occurrence was observed for AFs+FB_1_ and AFs+ZEN, which were detected in 100% of the rice bran samples, followed by AFs+OTA and AFs+DON at 99.2% and 93.6%, respectively. Furthermore, co-contamination of AFs+DON+OTA+FB_1_ was observed in 92.8% of the rice bran samples. In maize, AFs+FB_1_ and AFs+OTA, were the most frequent co-occurring mycotoxins at 96.8% and 90.4%, respectively. The combination of four regulated mycotoxins (AFs+DON+OTA+FB_1_) was observed in 37.6% of maize samples.

The co-occurring regulated and emerging mycotoxins found in rice bran and maize samples are presented in Figure 3. DON+BEA combination in rice bran and maize samples at 93.6% and 45.6%, respectively, whereas DON+ENN type B was present in rice bran and maize samples at 93.6% and 44.8%, respectively. In the case of ZEN, the combination of ZEN+BEA and ZEN+ ENN type B was present in rice bran samples at 100% and in maize samples at 64.0%.

**Figure 4:**
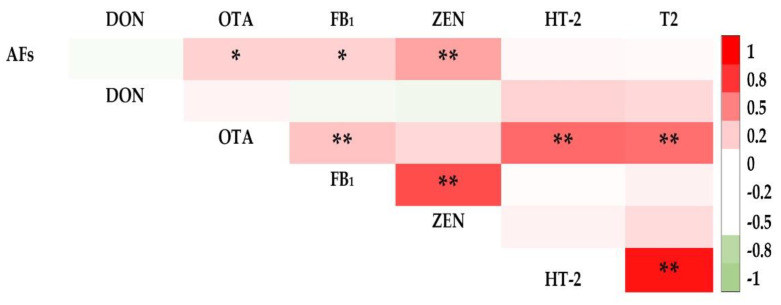
Pearson’s correlation coefficient for regulated mycotoxins in rice bran. **, and * represent *p*-values of 0.05, and 0.01, respectively.

**Figure 5:**
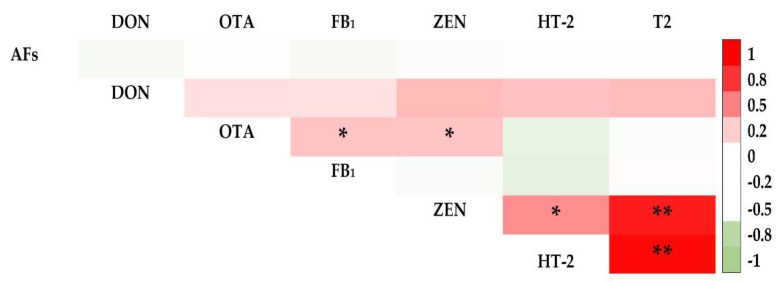
Pearson’s correlation coefficient for regulated in maize. **, and * represent *p*-values of 0.05, and 0.01, respectively.

In rice bran, the occurrence of FB_1_ was strongly correlated with ZEN (*r* = 0.702). HT-2 and T2 were also strongly correlated (*r* = 0.927) (Figure 4). Beauvericin was found to be strongly correlated with ZEN. Moreover, the occurrence of AFs was correlated with OTA, FB_1_, and ZEN (*r* = 0.181, 0.179, and 0.363, respectively) (Figure 4). In maize, strong correlations were found between T2 and ZEN (*r* = 0.898) and T2 and HT-2 (*r* = 0.971) (Figure 5).

## 3. Discussion

### 3.1. Climate Change and Mycotoxins

Mycotoxins are harmful fungal metabolites frequently found in cereal grains and feed products. They have been shown to negatively impact food and feed safety because of the potential carry-over of mycotoxins to animal by-products, including meat products and dairy products, leading to possible mycotoxin uptake by humans as a consequence of the animal eating contaminated feed [32]. The climate of Southeast Asia can be classified as mainly tropical and one of the most climate-vulnerable regions in the world. It has been identified as a region where predicted changes in temperature, CO_2_ levels, and rainfall patterns are exacerbated as a result of the changing climate [33]. The weather is hot and humid most of the year, which contributes to the proliferation of mycotoxin-producing fungi that release toxic metabolites and thus lead the way to food and feed contamination [34]. The main factors that boost mycotoxins’ fungal growth and production are temperature and humidity. A temperature range of 25 °C to 30 °C, a water activity (*a*_w_) of more than 0.78, and relative humidity (RH) of 88% to 95% are all regarded as suitable for the growth of a fungal and subsequent generation of mycotoxin [35]. The Intergovernmental Panel on Climate Change (IPCC) published a study in 2014 on global warming forecasts, estimating that global temperature might rise by up to 4.8 °C in the 21st century [36]. Climate change would directly impact agriculture, with changes in temperature and humidity affecting the efficacy of insecticides and fungicides, which may cause the adaption of some fungal species to generate new metabolites, which can cause a negative effect on humans and animals [37].

The studied samples were collected from Cambodia, Laos, Myanmar, and Thailand from August to December 2020. The average temperature range was 25 °C to 28 °C. Most of Southeast Asia experienced above-average rainfall between August and December; for example, the highest rainfall in Cambodia and Thailand was recorded in October of this year, while for Laos and Myanmar, the highest rainfall occurred during August [38,39]. Climatic conditions influence mycotoxin levels and incidences of contamination in crops year-to-year. For example, in Thailand, high levels of FBs in corn and ZEN in rice bran in 2011 were observed compared to 2010, 2012–2014 due to flooding in July 2011 [40]. Similarly, high levels of AFB_1_ in maize were found in Southeast Asia in 2017 compared to 2013–2016 due to high rainfall and temperature during the spiking period [2]. BIOMIN survey reported that the most prevalent mycotoxins in Southeast Asia in 2020 were FBs and AFs [41], with the results of this study in agreement with their finding. However, the level of mycotoxins in samples varies by commodity and country.

### 3.2. Occurrence of Regulated Mycotoxins in Rice Bran and Maize

The results of this study indicated that of the 250-rice bran and maize samples tested, 100% of them indicated the presence of AFs (AFB_1_, AFB_2_, AFG_1_, AFG_2_) with concentrations ranging between 0.1–2149.7 µg·kg^−1^. A prevalence of AFs was observed, with 100% of samples testing positive at values of 0.4–271.1 µg·kg^−1^ (mean level of 13.0 µg·kg^−1^) for rice bran and 0.1–2149.7 µg·kg^−1^ (mean level of 158.1 µg·kg^−1^) in maize with contamination in samples from Cambodia, Laos, Myanmar, and Thailand. The incidence of AFs, particularly AFB_1_, indicated that 2% of rice bran and 28% of maize samples positive for AFs were contaminated at a level higher than the European Commission’s maximum levels of 20 µg·kg^−1^ for feed materials [15].

Rice bran and maize are the main constituents of local animals’ diets, consumed in large quantities and supplemented directly or indirectly. As a result, AFs contamination could be dangerous even at low levels. Furthermore, the occurrence of mycotoxins in feedstuff is usually influenced by weather; as a result, the type of mycotoxin varies with time and geographical location, with the main factors influencing mould growth being temperature and humidity [42]. Consequently, Southeast Asia is a region with a high prevalence of AFs. Levels of AFs have been found in rice bran and maize for feed in the Philippines (2005), Vietnam (2008), and Thailand from 2015–2020 at levels ranging from 0.27–11 µg·kg^−1^ [6,43,44]. Therefore, it is recommended that storage conditions be improved to minimize spoilage and limit aflatoxins contamination.

In this study, FB_1_, ZEN, and OTA were found in samples from Cambodia, Laos, Myanmar, and Thailand. FB_1_ and ZEN were observed in 100% of rice bran samples at values of 61.5–7013.8 µg·kg^−1^ (mean level of 579.6 µg·kg^−1^) and 21.9–1728.4 µg·kg^−1^ (mean level of 183.2 µg·kg^−1^), respectively. The observed results indicated a higher prevalence than that reported by Kananub et al. [6]. The presence of FB_1_ and OTA was detected in over 90% of maize samples with concentrations ranging from 1.8–20900.9 µg·kg^−1^ (mean concentration of 2786.0 µg·kg^−1^) and 0.3–65.5 µg·kg^−1^ (mean concentration of 12.0 µg·kg^−1^), respectively. Previously, researchers found that the varieties of maize significantly impacted the occurrence of OTA [45]. Compared to the EU guidance limits [16], the detected levels of FB_1_, ZEN, and OTA were lower than the safety limits. Guidance values set for ZEN and OTA are 2000 µg·kg^−1^ and 250 µg·kg^−1^, respectively, in cereals and cereal products for feedstuff. In maize by-product, guidance levels of 3000 µg·kg^−1^, and 60000 µg·kg^−1^ were set for ZEN, and the sum of FB_1_ and FB_2_, respectively.

### 3.3. Occurrence of Emerging Mycotoxins in Rice Bran and Maize

Emerging mycotoxins are a new group of mycotoxins [46] that are not legislatively regulated. However, research shows that these toxins quickly become common co-contaminants in grains such as maize, wheat, and rice. As a result of monitoring and reporting BEA and ENNs have raised a lot of interest, with toxicity studies and risk assessments having been recently carried out [47,48]. In this study, BEA was detected with a high prevalence in rice bran and maize samples, while on the other hand, ENN type B and STC were detected with a high prevalence in rice bran.

BEA has been identified as being toxic in vitro to human tissues and cells [49]. BEA has cytotoxic effects on various cell types and can induce oxidative stress at the molecular level. In addition, it causes genotoxicity and apoptosis in the mitochondrial pathway. Although severe cytotoxicity has been reported, regulatory authorities have not performed a risk assessment due to a lack of toxicity data [50]. In the reported study, a high occurrence of BEA in rice bran and maize was observed, with concentrations ranging from 1.25–2281.01 µg·kg^−1^ (mean 130.37 µg·kg^−1^) and 0.02–1459.18 µg·kg^−1^ (mean 60.67 µg·kg^−1^), respectively. These levels were higher than those reported in studies performed in Japan, China, and Korea [51,52,53]. BEA was found in corn grits from the Japanese market at 26.1 µg·kg^−1^ [51]. An evaluation of commercial pet food in China revealed that 96.9% of samples tested were contaminated with BEA at concentrations ranging from 0.2–153.4 µg·kg^−1^ [52]. The distribution of feed materials and compound feed in Korea that contained BEA-contaminated was evaluated by Lee et al. [53]. The results highlighted that the percentage of samples tested contaminated with BEA was 27% at levels between 0.01–1.80 µg·kg^−1^. BEA has been observed in corn-dried distiller grains in Thailand (350 µg·kg^−1^), Irish farm silages (21.8 µg·kg^−1^), and rice bran from Spain (64.8 µg·kg^−1^) [54,55,56]. Furthermore, a survey in the EU revealed that 98% (*n* = 83) of feed and raw feed materials analysed were contaminated with BEA, with a maximum level of 2326 g·kg^−1^ [57].

Concerning ENNs type B, this study revealed that this mycotoxin was present in over 95% of rice bran samples analysed. Like BEA, ENNs cause cytotoxicity and genotoxicity. Both ENNs and BEA are cyclic hexadepsipeptides with similar biological pathways [46], however, due to limited toxicity data, a risk assessment has not been established. Our findings highlighted concentrations of ENN B ranging between 0.11–15.36 µg·kg^−1^ (mean 6.73 µg·kg^−1^), whereas ENN B_1_ levels ranged between 0.53-14.70 µg·kg^−1^ (mean 2.58 µg·kg^−1^), with rice bran indicating higher levels than maize for these two mycotoxins. These results are higher than those found by Shimshoni et al. [58] from Israel, where mean values of 0.3 µg·kg^−1^ and 0.9 µg·kg^−1^, respectively, were detected in both corn and wheat samples. Jestoi et al. [59] reported the occurrence of ENN B and ENN B_1_ in barley samples from Finland, at 3980 µg·kg^−1^ and 3240 µg·kg^−1^, respectively. According to Uhlig et al. [60], ENN B was detected in wheat samples from Norway at a maximum concentration of 5800 µg·kg^−1^, and Sorensen et al. [61] reported contaminations levels of ENN B in whole fresh maize in Denmark of 2600 µg·kg^−1^. Monitoring of wheat in Romania for the presence of ENNs. Researchers reported the occurrence of ENN B in wheat and wheat flour, at 21–407 µg·kg^−1^ (71% of positive samples) [62] indicating that the higher the humidity, the greater the contamination of ENNs [62,63]. In such cooler climates, the conditions are optimal for the proliferation of *Fusarium* spp. Therefore, this explains the higher concentrations of ENNs in the countries mentioned [56]. This study highlighted significant levels BEA and ENN type B in rice brans (100% of samples); thus, the co-occurrence of the emerging *Fusarium* mycotoxins should be closely monitored particularly because of the possible synergistic effects of these mycoestrogens [56,64].

The occurrence of STC has been found in many types of food and feed [65,66,67,68,69]. STC is a precursor of AFs in their biosynthesis and is known to be much less toxic than AFB_1_. Its hepatotoxicity and nephrotoxicity have been demonstrated in animals however, due to the limit of information on toxicity, the authorities have not prepared a risk assessment [70]. In our study, STC was more often found in rice bran than in maize. It was quantifiable in 97% (122 of 125) samples, with a mean concentration of 26.63 µg·kg^−1^. This is probably a result of the proliferation of toxin-producing fungi at the aleurone layer of rice bran [5]. Previously, some derivatives of the toxin have been reported. The incidence of STC in Italian paddy rice was detected, and on the removal of the bran layer, the levels of STC were significantly reduced. This indicates that rice processing causes a reduction of STC [71]. Although STC is considered by the International Agency for Research on Cancer (IARC) as a group 2B, carcinogenic to animals and possibly humans also, the European legislation has no set limits for STC in food; however, the Czech Republic and Slovakia have set limits of STC for rice, poultry, meat, milk, vegetables, potatoes, and flour at 5 µg·kg^−1^ and other foods at 20 µg·kg^−1^ [71].

### 3.4. Co-Occurrence of Regulated Mycotoxins in Rice Bran and Maize

There are limited data on the combined toxic effects of mycotoxins, therefore the health risks from multiple mycotoxin exposure are unknown. The natural prevalence of mycotoxins in food and feed is quite not unusual. Some fungi can produce more than one mycotoxin, multiple fungi may infect agricultural commodities, and the influence of climate will have a huge impact on contamination levels [3]. The study of combined toxicological effects such as antagonist, additive, or synergic effects is difficult to predict and may be a significant threat to human and animal health; therefore, the toxicological interactions between mycotoxins must not be overlooked [72]. The co-occurrence with other mycotoxins and AFs should be investigated. There has been reported the occurrence of AFs contamination in rice with a high prevalence in many Asian countries [73]. In this study, contamination with at least two or more mycotoxins was frequently observed. Although risk analysis is performed on a single mycotoxin to perform the risk assessment of carcinogenic agents rather than multiple mycotoxins, it is important to consider the cumulative toxicity of multiple mycotoxins given that these toxins rarely occur on their own.

In the study, the samples contained at least two mycotoxins, and the co-occurrence of AFs+FB_1_ was higher than in the study in South America, which reported co-occurrence of AFs and FBs in maize at 7–8% [74,75] and 54.5% of AFB_1_+FB_1_ [76]. The combined action of mycotoxins can have an interaction effect. Aflatoxin-fumonisin co-exposure has been shown to have additive or synergistic effects on the expression of precancerous lesions or liver cancer in several in vitro investigations [77]. The co-occurrence of AFs+OTA in this study was higher than in the study of Ibáñez-Vea et al. [78] in which detectable levels of AFs+OTA were observed in 31% of barley samples from Spain. It has been demonstrated that the combination AFs+OTA established a synergistic effect on nephrotoxicity [79]. Previously, the combination AFs+DON was observed in soy feed samples [80], and again the observed results were lower than those observed in this study. This is important as previous research has highlighted synergistic effects associated with this combination of mycotoxins [81,82].

Moreover, numerous surveys have revealed the co-occurrence of mycotoxins involving more than two mycotoxins. In 2016, DON, fumonisin (FB_1_ and FB_2_), ZEA, and the trichothecenes, T-2 and HT-2 were found in 26.2% of maize samples in Croatian fields [83]. Palumbo et al. [84] reported that DON frequently co-occurred with FBs in maize (74.4%), whereas the incidence of DON, FBs, and AFs was found to be low (1.0%). The combinations of mycotoxins observed in our investigation were generally consistent with those reported by other researchers in previous studies [76,78,80].

Data on the effect of climate change on the co-occurrence of mycotoxins are limited. However, climate change may indirectly impact the levels of co-occurring mycotoxins contamination. Co-occurrence of mycotoxins has been proven to be exceedingly toxic and influence feed intake; hence this warrants more attention [85]. It is well known that the incidences and contamination levels of mycotoxins depend on the harvest year. Changing weather (i.e., temperature and rainfall) is the most important climatic factor that will impact mycotoxin contamination in crops in Southeast Asia in the future [86,87]. Increases in temperature by 1 or 2 °C (above-average temperature) or decreases in rainfall would ultimately reduce production yield. These changing conditions will influence the proliferation of specific fungi and, consequently, the production of mycotoxins [34]. Therefore, it is essential to monitor the co-occurrence of mycotoxins and perform risk assessments to protect the human and animal populations against increased toxic effects due to the possible synergistic interactions of mycotoxins [88].

### 3.5. Co-Occurrence of Regulated and Emerging Mycotoxins in Rice Bran and Maize

The natural co-occurrence of the regulated mycotoxins has been reported in several studies, particularly, AFs, DON, FBs, OTA, and ZEN. Fewer data have been reported for the contamination of the regulated mycotoxins and emerging mycotoxins (i.e., BEA, ENNs, and Alternaria toxins). DON and ZEN were assessed in this investigation because it is one of the most highly distributed contaminants in feed. Exposure to high levels of these toxins is associated with function in animals; as an example, the chronic levels of DON and ZEN affect the kidney function in swine that is more sensitive to exposure to DON and ZEN than most other species [89]. Consumption of feed contaminated with DON can lead to vomiting, diarrhoea, refusal of feed, and weight loss of animals. ZEN is an estrogenic mycotoxin that adversely affects reproductive function in animals [89]. Previously, the combined toxicity of DON, BEA, and ENN type B was studied. According to Perez-Fuentes et al. [90], the combination of DON and emerging mycotoxins mixtures exhibited antagonism when used to treat neuroblastoma cells [91].

Moreover, in the same study by Perez-Fuentes et al. [90], the combination of ZEN and emerging mycotoxins (ENNs and BEA) also demonstrated an antagonistic effect. Although the contamination of regulated mycotoxins and emerging mycotoxins in samples has been widely documented, there has been limited research on their combined toxicity. Nonetheless, the current regulations do not consider the impacts of many mycotoxins, and the maximum limit allowed, or recommended levels are set for a single mycotoxin.

The overall results show that the mycotoxins affecting rice crops and maize in Southeast Asia include AFs, FBs, DON, OTA, and ZEN as well as the emerging mycotoxins BEA, ENNs, and STC. Mycotoxin contamination of Southeast Asia crops will undoubtedly increase due to climate changes, including higher temperatures, increased rainfall, drought, and floods. Most of Southeast Asia has already experienced above-average temperature and above-average rainfall. According to global climate change scenarios, the global temperature will rise by up to 4.8 °C in the 21st century [36]. In addition, climate change has been shown to affect the population of toxigenic fungi and mycotoxin levels in crops. Therefore, the mycotoxin problem and toxic effects of combined mycotoxins should be carefully monitored as they are potentially a significant threat to human and animal health. Hence, it is incumbent on governments and/or research institutions in each country to try to improve farming systems and/or techniques to minimize the effects of climate change in the future and create awareness of mycotoxin control, good storage, and distribution practices to reduce the production of mycotoxins in agricultural crops.

### 3.6. Mycotoxin Control Strategies

Factors such as poor-quality control, poor production technology, and poor crop storage condition encourage the growth of fungi and mycotoxin formation in developing countries, resulting in a higher prevalence of mycotoxin-contaminated foods [92,93]. Crops that are harvested early and dried can help to reduce contamination. However, in several developing countries, early harvesting, uncertain weather, labour constraints, a need for returns on their crops, and the threat of animals compel farmers to harvest at inappropriate times. Strategies for prevention are a key approach in the fight against mycotoxins, including good agricultural practices (GAPs), good manufacturing practices (GMPs), hazard analysis and critical control points (HACCP), and appropriate storage conditions in terms of moisture and temperature control. Among the environmental factors, temperature and humidity majorly impact mycotoxigenic fungi to produce mycotoxins. Therefore, temperature, moisture content, and humidity of warehouse storage are critical factors for fungal growth and mycotoxin production [94].

## 4. Conclusions

The regulated mycotoxins, emerging mycotoxins, and masked mycotoxins were analysed in rice bran and maize samples from four Southeast Asia countries, Cambodia, Laos, Myanmar, and Thailand. The most prevalent mycotoxins in Southeast Asia from August to December 2020 were Afs, FBs, DON, OTA, and ZEN, and the emerging mycotoxins (BEA, ENNs, and STC). AFs were detected in 100% of rice bran and maize samples. The difference in mycotoxin occurrence has been highlighted in the different sampling zones, the detection levels of AFs in rice bran were highest in samples sourced from Myanmar (Myanmar>Laos>Thailand>Cambodia) whereas the levels in maize were highest in samples from Laos (Laos>Cambodia>Myanmar>Thailand). FB_1_, ZEN, OTA, DON, BEA, ENN type B, and STC were detected in greater than 90% of samples. This study demonstrates mycotoxin levels in all samples in compliance with EU regulations (except AFs and one sample for HT-2). Moreover, the results showed co-occurrence of *Aspergillus* and *Fusarium* mycotoxins were most commonly found in feed samples with a high prevalence of co-occurring of regulated mycotoxins (DON and ZEN) with emerging mycotoxins (BEA and ENN type B).

## 5. Materials and Methods

### 5.1. Sample Collection

A total of 125 rice bran and 125 maize samples, commonly used to formulate feed to meet the energy requirements of livestock animals were collected from major feed mills in Cambodia, Laos, Myanmar, and Thailand, from August to December 2020. Each aggregate sample had approximately 1 kg and was collected according to the European Commission Regulation (EC) No 152/2009 guidelines. All samples were ground into powder using a Tecator Cyclotec 1093 mill fitted with a 1 mm sieve (Foss, Denmark), then stored at −20 °C before mycotoxin analysis.

### 5.2. Sample Preparation

Sample extraction was performed using a dilute-and-shoot approach [95,96], with 1 g of homogenised sample weighed into a 15-mL polypropylene tube. Next, 4 mL of acetonitrile: water: formic acid (79:20:1, *v*/*v*/*v*) was added, and the sample was vortexed for 90 min. After centrifugation at 5000 rpm for 15 min, an aliquot of 250 µL of the supernatant was mixed with 750 µL of acetonitrile: water: formic acid (20:79:1, *v*/*v*/*v*) in an Eppendorf tube. The mixture was vortexed for 30 s and filtered through a 0.2 µm PTFE syringe filter into an LC-MS/MS vial for analysis.

### 5.3. Chemicals and Materials

Ammonium hydroxide (≥25% in water), LC−MS grade methanol, and acetonitrile were obtained from Sigma-Aldrich (Gillingham, UK). A Milli-Q system (Millipore, Molsheim, France) was used as a source of deionized water. Mycotoxins standards: aflatoxin B_1_ (AFB_1_), aflatoxin B_2_ (AFB_2_), aflatoxin G_1_ (AFG_1_), aflatoxin G_2_ (AFG_2_), alternariol, beauvericin (BEA), deoxynivalenol (DON), enniatin A (ENN A), enniatin A_1_ (ENN A_1_), enniatin B (ENN B), enniatin B_1_ (ENN B_1_), fumonisin B_1_ (FB_1_), HT-2 toxin (HT-2), ochratoxin A (OTA), T2 toxin (T-2), and zearalenone (ZEN) were obtained from Romer lab, UK. Ergocornine (Ergocor), ergocristine (Ergocris), sterigmatocystin (STC), and stachybotrylactam (STLAC) were obtained from Sigma-Aldrich (Gillingham, UK). 3-Acetyl-deoxynivalenol (3-Acetyl-DON), 15-Acetyl-deoxynivalenol (15-Acetyl-DON), deoxynivalenol-3-glucoside (DON-3-Glu), zearalenone-14-glucoside (ZEN-14-G) and zearalenone-16-glucoside (ZEN-16-G) were obtained from Enzo science (Exeter, UK). From the solid standards, individual stock solutions were prepared at a 1 mg·mL^−^^1^ concentration in an appropriate solvent and stored based on the manufacturer’s instructions. All standard solutions were kept in amber glass vials at −20 °C and brought to room temperature before use. The stock solutions were renewed every 2 months, while multi-mycotoxin working solutions were prepared weekly.

### 5.4. HPLC-MS/MS Parameters

Quantitative analysis of the targeted mycotoxins in each feed raw materials was carried out on an ExionLC™ AD Ultra-High Performance Liquid Chromatography system (SCIEX, Framingham, MA, USA) coupled with a SCIEX 5500+ QTrap triple quadrupole Mass Spectrometer (MS/MS) equipped with a Turbo V™ electrospray ionisation source (SCIEX, Framingham, MA, USA). Chromatographic separation was achieved using a Gemini C18-column (100 × 4.6 mm, 5,μm) maintained at 30 °C. Elution was carried out in a binary gradient mode consisting of mobile phase A—methanol/water/acetic acid 10:89:1 (*v*/*v*/*v*) and mobile phase B—methanol/water/acetic acid 97:2:1 (*v*/*v*/*v*), both containing 5 mM ammonium acetate buffer. Mycotoxins were eluted following a gradient elution program as follows: 0 min 1% B, held for 1 min at 1% B, 5 min 65% B, 7 min 80% B, 8.5 min 80% B, 9 min 95% B, 10 min 95% B and 11.5 min 1% B. A mobile phase flow rate was maintained at 0.7 mL·min^−1^, with sample injection volume set at 3 µL, and the total runtime was 11.5 min.

The Mass Spectrometry was operated in both positive and negative electrospray ionisation mode, with data acquisition carried out in scheduled multiple reaction monitoring (sMRM) mode. The capillary voltage and source temperatures were set at 4.5 kV and −4.5 kV for ESI+ and ESI- respectively, with the temperature set at 600 °C. Collision gas, ion source gas (GS1), ion source gas (GS2), and curtain gas were set at 9, 60, 60, and 35 psi, respectively. Two MRM characteristic transitions (1 precursor ion, 2 product ions) were monitored for each analyte. The selected MRM transitions and their respective analyte-dependent operating conditions, i.e., declustering potential (DP), collision cell exit potential (CXP), and collision energy (CE), are listed in Table 1. Analyst® Software 1.7.1 and SCIEX OS-Q Software were used for acquiring and processing data, respectively.

### 5.5. Method Validation

The optimised LC-MS/MS method for the analysis of mycotoxins in rice bran and maize was validated based on the acceptable performance criteria of analytical methods set by the European Commission regulations No. 2002/657/EC [97]. The performance characteristics evaluated were linearity, limit of detection (LOD), limit of quantification (LOQ), selectivity, matrix effect, recovery, and repeatability.

Extraction and apparent recoveries were determined by spiking homogenised feed raw materials at three different levels with a multi-mycotoxin standard solution (i.e., low (10 ng·g^−1^), medium (50 ng·g^−1^), and high (200 ng·g^−1^). Spiked samples were placed in the dark overnight to allow evaporation of the solvent and interaction of the analyte with the matrix. The spiked and blank (control or unspiked) samples were extracted as described above. Following sample extraction, blank samples were also spiked with the same levels of multi-mycotoxin solution to determine the extraction efficiency/recovery. Recovery of each analyte was calculated as the ratio of peak area of blank sample spiked before and after sample extraction multiplied, expressed as a percentage (%). Matrix-induced suppression/enhancement (SSE) was determined by comparing the response of the matrix spiked with standards after extraction to a solvent standard at the same concentration. SSE was calculated as the ratio of the peak area of the analyte in the matrix and solvent, expressed as a percentage (%).

(1) $$SSE=\frac{\mathrm{Peak}\;\mathrm{area}\;\mathrm{of}\;\mathrm{analyte}\;\mathrm{in}\;\mathrm{matrix}}{\mathrm{Peak}\;\mathrm{area}\;\mathrm{of}\;\mathrm{analyte}\;\mathrm{in}\;\mathrm{solvent}}\times 100$$

Linearity was evaluated using mycotoxin-free samples fortified with multi-mycotoxin working solutions at seven concentration levels. The spiked samples’ concentration range was chosen to cover the estimated linear range of calibration, levels commonly found in naturally contaminated samples and legislation limits of regulated mycotoxins, and the respective limits of detection (LOD) of each analyte. LODs and LOQs determined the sensitivity of the developed method. LOD was defined as the concentration of each analyte that gave a peak with a signal-to-noise ratio (S/N) of 3, which was determined by injecting neat solvent standard solution at different concentrations, while LOQ was defined as the concentration of the analyte in spiked samples at an S/N ratio of 10. Precision was determined by intra-day precision (repeatability) and inter-day precision (reproducibility). Intra-day precision was carried out by analysis of three replicates on the same day at three different concentration levels, while inter-day precision was assessed by repeating the same procedure over three consecutive days. The data were used to calculate within-laboratory accuracy and precision and expressed as relative standard deviation (RSD). The criteria for confirming a positive sample include a retention time within ±0.5% compared with the analyte in a pure solvent, both qualifier and the quantifier with transitions above S/N ratio of 10:1, and the ion ratio of the quantifier and the qualifier transition within ±25%.

### 5.6. Statistical Analyses

The Pearson’s correlation coefficient was used to calculate the correlation; data with *p <* 0.05 and *p <* 0.01 values were considered statistically significantly different using the SPSS 10.0 software (SPSS, Chicago, IL, USA).

## Figures and Tables

**Table 1:** Optimised MS/MS parameters for the analysed compounds, including precursor ions, product ions, declustering potential (DP), collision energy (CE), and collision cell exit potential (CXP).

Mycotoxins	Precursor ion (*m*/*z*)	Product ion (*m*/*z*)	DP (V)	CE (eV)	CXP
**Regulated mycotoxins**					
Aflatoxin B_1_	313.061	285.1	121	33	14
	313.061	241.1	121	53	14
Aflatoxin B_2_	315.074	287.2	141	37	14
	315.074	259.1	141	41	14
Aflatoxin G_1_	329.055	243.2	131	37	18
	329.055	311.1	131	31	16
Aflatoxin G_2_	331.057	313	106	35	16
	331.057	245.2	106	41	14
Deoxynivalenol	297.097	249.1	91	21	20
	297.097	203.2	91	21	20
Fumonisin B_1_	722.316	334.4	100	53	10
	722.316	704.3	100	41	38
HT-2 toxin	447.169	345.1	131	27	18
	447.169	285.2	131	29	14
T2 toxin	489.175	387.2	151	31	36
	489.175	245.2	151	35	12
Ochratoxin A	404.092	239	111	33	12
	404.092	358.1	111	21	18
Zearalenone	319.114	301.1	81	15	16
	319.114	283.1	81	17	14
**Emerging mycotoxins**					
Alternariol	256.957	213	−125	−34	−19
	256.957	215	−125	−36	−17
Beauvericin	801.287	784.3	141	27	14
	801.287	244.1	141	43	12
Enniatin A	699.386	682.4	100	27	24
	699.386	210.2	100	39	22
Enniatin A_1_	685.36	668.5	100	27	12
	685.36	210.1	100	39	10
Enniatin B	657.319	640.3	100	27	22
	657.319	196.1	100	39	10
Enniatin B_1_	671.317	654.4	6	27	22
	671.317	196.1	6	41	22
Stachybotrylactam	386.184	178	191	47	22
	386.184	150.2	191	57	14
Sterigmatocystin	325.023	310.1	121	35	16
	325.023	281.6	121	41	14
**Masked mycotoxin**					
3-acetyl deoxynivalenol	337.1	59.2	−110	−28	−13
	337.1	255.2	−110	−52	−9
15-acetyl deoxynivalenol	339.1	321.3	81	13	18
	339.1	261.1	81	17	14
Deoxynivalenol-3-glucoside	517.3	427.1	−70	−30	−11
	517.3	59.1	−70	−85	−7
Alpha-Zearalenol	319.2	160.1	−105	−44	−13
	319.2	130.1	−105	−50	−20
Beta-Zearalenol	319.2	160	−105	−44	−13
	319.2	130	−105	−50	−20
Zearalenone-14-glucoside	479.2	317.1	−145	−24	−15
	479.2	175	−145	−54	−11
Zearalenone-14-glucoside	479.12	317.105	−140	−30	−21
	479.12	149	−140	−50	−15

## Data Availability

Not applicable.

## Supplementary Materials

The following supporting information can be downloaded at: https://www.mdpi.com/article/10.3390/toxins14080567/s1, Table S1: The EU guidance levels for AFB1 and recommended limits for some regulated mycotoxins in feed materials; Table S2: Linearity data (R2), limit of detection (LOD), and limit of quantification (LOQ) of mycotoxins in the analysed rice bran and maize using LC-MS/MS; Table S3: Recovery and precision of mycotoxins in the analysed rice bran and maize by LC-MS/MS; Table S4: The occurrence and concentration of mycotoxins in rice bran and maize.
