# Supplementary material for: The Occurrence and Co-Occurrence of Regulated, Emerging, and Masked Mycotoxins in Rice Bran and Maize from Southeast Asia
Source: Toxins (Basel). 2022 Aug 19;14(8):567. doi: 10.3390/toxins14080567 (PMC9412313; doi:10.3390/toxins14080567)
Supplement: Supplementary file 1 [file toxins-14-00567-s001.zip › toxins-1860175-supplementary.pdf]

Supplementary Materials: The Occurrence and Co-occurrence of Regulated, Emerging and Masked Mycotoxins in Rice Bran and Maize from Southeast Asia

Wipada Siri-anusornsak , Oluwatobi Kolawole , Warapa Mahakarnchanakul [1], Brett Greer , Awanwee Petch kongkaew, Julie Meneely , Christopher Elliott and Kanithaporn Vangnai,*

Table S1. The EU guidance levels for AFB1 and recommended limits for some regulated mycotoxins in feed materials.

Mycotoxins	Matrix	Limits (μg.kg^{-1})	References
AFB_1	All feed materials	20	European Commission, 2002. [15]
The sum of FB_1 and FB_2	Maize and maize products	60,000	Commission Recommendation, 2006. [16]
ZEN	Cereals and cereal products<br>Maize by-products	2,000<br>3,000	Commission Recommendation, 2006. [16]
OTA	Cereals and cereal products	250	Commission Recommendation, 2006. [16]
DON	Cereals and cereal products<br>Maize by-products	8,000<br>12,000	Commission Recommendation, 2006. [16]

Table S2. Linearity data (R^2), limit of detection (LOD), and limit of quantification (LOQ) of mycotoxins in the analysed rice bran and maize using LC-MS/MS.

Mycotoxins	Linearity (R^2)		LOD (μg.kg^{-1})		LOQ (μg.kg^{-1})	
	Rice Bran	Maize	Rice Bran	Maize	Rice Bran	Maize
**Regulated mycotoxins**						
Aflatoxin B_1	0.9913	0.9973	0.1	0.05	0.5	1.0
Aflatoxin B_2	0.9904	0.9964	0.1	0.04	0.5	1.0
Aflatoxin G_1	0.9844	0.9924	0.1	0.04	0.5	1.0
Aflatoxin G_2	0.9921	0.9921	0.1	0.04	0.5	1.0
Deoxynivalenol	0.9911	0.9921	1.25	1.25	5.0	5.0
Fumonisin B_1	0.9922	0.9952	1.50	1.50	5.0	5.0
HT-2 toxin	0.9943	0.9943	1.00	1.00	5.0	5.0
T-2 toxin	0.9996	0.9906	1.00	1.00	5.0	5.0
Ochratoxin A	0.9943	0.9943	0.50	0.50	1.5	1.5
Zearalenone	0.9912	0.9962	1.50	1.50	5.0	5.0
**Emerging mycotoxins**						
Alternariol	0.9911	0.9927	0.05	0.05	3.5	3.5
Beauvericin	0.9921	0.9921	0.05	0.05	1.0	1.0
Enniatin A	0.9917	0.9967	0.05	0.05	5.0	5.0
Enniatin A_1	0.9934	0.9934	0.05	0.05	2.5	2.5
Enniatin B	0.9954	0.9954	0.05	0.05	2.5	2.5
Enniatin B_1	0.9935	0.9935	0.05	0.05	2.5	2.5
Ergocornine	0.9912	0.9942	0.50	0.50	2.5	2.5
Ergocristine	0.9911	0.9941	0.50	0.50	2.5	2.5
Stachybotrylactam	0.9902	0.9952	1.50	1.50	5.0	5.0
Sterigmatocystin	0.9931	0.9931	0.50	0.50	2.5	2.5
**Masked mycotoxins**						
3-acetyl deoxynivalenol	0.9917	0.9917	25.0	25.0	50.0	50
15-acetyl deoxynivalenol	0.9916	0.9966	5.0	5.0	20.0	20
Deoxynivalenol-3-glucoside	0.9941	0.9911	10.0	10	50.0	50
Alpha-Zearalenol	0.9951	0.9948	0.05	5.0	3.5	10

Beta-Zearalenol	0.9932	0.9932	0.05	5.0	3.5	10
Zearalenone-14- glucoside	0.9926	0.9956	0.50	10.0	2.5	50
Zearalenone-16- glucoside	0.9934	0.9902	0.50	10.0	2.5	50

Table S3. Recovery and precision of mycotoxins in the analysed rice bran and maize by LC-MS/MS.

Mycotoxins	Extraction Recovery (%)		RSD_r* (%)		RSDR** (%)	
	Rice Bran	Maize	Rice Bran	Maize	Rice Bran	Maize
**Regulated mycotoxins**						
Aflatoxin B_1	90	97	12	9	6.9	3.4
Aflatoxin B_2	85	96	10	11	4.1	2.6
Aflatoxin G_1	86	104	8	5	3.8	2.7
Aflatoxin G_2	86	98	7	7	5.5	3.6
Deoxynivalenol	82	75	8	5	13.1	7.2
Fumonisin B_1	76	80	12	15	8.9	6.1
HT-2 toxin	80	79	10	9	1.9	2.3
T-2 toxin	75	82	11	4	1.6	2.9
Ochratoxin A	85	95	10	8	4.6	2.8
Zearalenone	85	82	6	6	6.2	4.0
**Emerging mycotoxins**						
Alternariol	85	89	8	5	7.8	6.0
Beauvericin	80	80	7	8	8.5	6.0
Enniatin A	90	97	13	5	6.9	8.7
Enniatin A_1	85	86	8	11	5.9	4.4
Enniatin B	85	94	8	7	7.8	9.0
Enniatin B_1	88	88	12	10	9.9	3.5
Ergocornine	77	91	7	4	4.9	4.8
Ergocristine	77	98	10	10	10.6	3.9
Stachybotrylactam	91	91	5	6	5.1	3.0
Sterigmatocystin	92	105	4	5	7.0	8.9
**Masked mycotoxins**						
3-acetyl deoxynivalenol	80	82	9	5	4.6	8.7
15-acetyl deoxynivalenol	86	85	6	5	8.9	13.4
Deoxynivalenol-3-glucoside	86	85	7	5	14.4	8.5
Alpha-Zearalenol	75	87	3	10	4.5	3.9
Beta-Zearalenol	76	86	13	15	4.6	6.1
Zearalenone-14- glucoside	71	78	5	10	5.1	9.3
Zearalenone-16-glucoside	72	79	4	10	7.0	8.1

* RSDr for repeatability, ** RSDR for reproducibilit

Table S4. The occurrence and concentration of mycotoxins in rice bran and maize.

Mycotoxins	Rice Bran (N = 125)					Maize (N = 125)				
	Positive Sample (n)	% Prevalence	Concentration ($\mu g.kg^{-1}$)			Positive Sample (n)	% Prevalence	Concentration ($\mu g.kg^{-1}$)		
			Min	Max	Mean			Min	Max	Mean
**Regulated mycotoxins**										
Aflatoxins (AFB_1, AFB_2, AFG_1, AFG_2)	125	100	0.4	271.1	13.1	125	100	0.1	2149.7	159.4
Fumonisin B_1	125	100	61.5	7013.8	579.6	121	97	1.8	20900.9	2786.0
Zearalenone	125	100	21.9	1728.4	183.2	81	65	2.2	751.3	29.4
Ochratoxin A	124	99	1.8	43.7	20.1	113	90	0.3	65.5	12.0
Deoxynivalenol	117	94	0.1	218.9	56.0	57	45	0.6	598.3	31.8
HT-2 toxin	102	82	1.8	23.0	18.0	36	29	1.7	9786.0	283.6
T2 toxin	84	67	0.7	7.9	6.7	40	32	0.7	61.8	7.3
**Emerging mycotoxins**										
Beauvericin	125	100	1.3	2281.0	130.4	116	93	0.03	1459.2	61.2
Enniatin B	125	100	0.1	15.4	2.6	124	99	-	0.03	-
Enniatin B_1	125	100	0.5	14.7	4.6	122	98	0.03	0.8	0.04
Sterigmatocystin	122	98	2.8	272.3	26.6	54	43	0.3	17.9	8.8
Stachybotrylactam	99	79	1.5	29.5	15.5	12	10	0.8	15	9.5
Alternariol	50	40	0.03	0.8	0.1	88	70	0.03	3.5	0.1
**Masked mycotoxins**										
zearalenone-14-glucoside	75	60	1.3	20.7	10.5	3	2	0.8	9.3	3.7
zearalenone-16-glucoside	70	56	1.0	39.5	12.9	1	0.8	-	5.0	-
